# Coping Styles, Postpartum Depression, and Anxiety in Romanian Women: A Cross-Sectional Study Using the Brief COPE Inventory

**DOI:** 10.3390/jcm15031029

**Published:** 2026-01-27

**Authors:** Nadica Motofelea, Radu Galis, Florin Adrian Szasz, Alexandru Catalin Motofelea, Teodora Hoinoiu, Sorin Trinc, Ion Papava, Flavius Olaru, Costin Berceanu, Raluca Parvanescu, Maja Vilibić, Irma Pljakić, Andreea Crintea, Florica Voita-Mekeres, Dan-Bogdan Navolan

**Affiliations:** 1 Doctoral School, “Victor Babes” University of Medicine and Pharmacy Timisoara, 300041 Timisoara, Romania; nadica.motofelea@umft.ro; 2 Department of Obstetrics and Gynecology, “Victor Babes” University of Medicine and Pharmacy Timisoara, Eftimie Murgu Square No. 2, 300041 Timisoara, Romania; olaru.flavius@umft.ro (F.O.); raluca.reitmayer@umft.ro (R.P.); navolan@umft.ro (D.-B.N.); 3 Department of Clinical Practical Skills, “Victor Babes” University of Medicine and Pharmacy Timisoara, 300041 Timisoara, Romania; tstoichitoiu@umft.ro (T.H.); irma.pljakic95@gmail.com (I.P.); 4 Department of Medical Sciences, Faculty of Medicine and Pharmacy, Oradea University, 410087 Oradea, Romania; 5 Department of Obstetrics Gynecology, Faculty of Medicine and Pharmacy, Oradea University, 410087 Oradea, Romania; 6 Center of Molecular Research in Nephrology and Vascular Disease, “Victor Babes” University of Medicine and Pharmacy Timisoara, Eftimie Murgu Square No. 2, 300041 Timisoara, Romania; alexandru.motofelea@umft.ro; 7 Center for Advanced Research in Cardiovascular Pathology and Hemostaseology, “Victor Babes” University of Medicine and Pharmacy Timisoara, 300041 Timisoara, Romania; 8 Department of Microscopic Morphology, Genetics Discipline, Center of Genomic Medicine, “Victor Babes” University of Medicine and Pharmacy Timisoara, 300041 Timisoara, Romania; sorin.trinc@umft.ro; 9 Department of Psychiatry, “Victor Babes” University of Medicine and Pharmacy Timisoara, 300041 Timisoara, Romania; papava.ion@umft.ro; 10 Department of Obstetrics Gynecology, University of Medicine and Pharmacy, 200349 Craiova, Romania; costin.berceanu@umfcv.ro; 11 Department of Psychiatry, Sestre Milosrdnice University Hospital Center, 10000 Zagreb, Croatia; maja.vilibic@gmail.com; 12 School of Medicine, Catholic University of Croatia, 10000 Zagreb, Croatia; 13 Department of Molecular Sciences, University of Medicine and Pharmacy “Iuliu Hațieganu”, 400349 Cluj-Napoca, Romania; crintea.andreea@umfcluj.ro; 14 Department of Morphological Disciplines, Faculty of Medicine and Pharmacy, University of Oradea, 410087 Oradea, Romania; voita-mekeres.f@uoradea.ro

**Keywords:** anxiety, COPE, coping, EPDS, GAD-7, PHQ-9, postpartum depression, postpartum women, religiosity, Romania

## Abstract

**Background/Objectives:** Postpartum depression and anxiety affect up to 20% of women worldwide, yet remain understudied in Eastern Europe. Romania, one of Europe’s most religious countries, provides a unique context for examining how coping strategies and religiosity influence perinatal mental health. This cross-sectional study characterized coping styles, depressive and anxiety symptoms, and religiosity among postpartum women from two western Romanian counties (Bihor and Timiș) and examined associations between coping dimensions and psychological outcomes. **Methods:** A cross-sectional study was conducted among 201 postpartum women recruited from two public maternity hospitals between 2024 and 2025. Sociodemographic, obstetric, neonatal, coping (COPE Inventory), depressive (EPDS; PHQ-9), anxiety (GAD-7), and religiosity (RCI-10) data were collected through questionnaires and medical record extraction. Associations between coping dimensions and psychological symptoms were examined using Pearson correlations. Analyses were performed in RStudio. **Results:** Prevalence of possible depression (EPDS ≥ 10) was 32.8% overall, with no regional difference (Bihor 32.0% vs. Timiș 33.7%, *p* = 0.920). EPDS demonstrated strong convergent validity with PHQ-9 (r = 0.58, *p* < 0.001) and GAD-7 (r = 0.61, *p* < 0.001). In bivariate analyses, avoidant coping showed the strongest association with depressive symptoms (r = 0.28, *p* < 0.001), particularly in Bihor (r = 0.35, *p* < 0.001). Multiple regression analysis (R^2^ = 0.196, *p* < 0.001) identified avoidant coping as the strongest independent predictor (β = 2.82, 95% CI [1.16, 4.48], *p* < 0.001), followed by social support coping (β = 2.46, 95% CI [1.09, 3.83], *p* < 0.001). Emotion-focused coping showed an unexpected protective effect (β = −2.97, *p* = 0.004). Problem-focused coping and religiosity were not significant predictors. Critically, county was non-significant after controlling for coping strategies (*p* = 0.732), indicating regional differences are mediated by coping patterns rather than geographic location. **Conclusions:** Postpartum depression prevalence in Romania aligns with international estimates. Avoidant coping emerged as the primary modifiable risk factor. Findings support integrating coping assessment into postpartum screening and developing interventions targeting avoidant strategies in Romanian perinatal care.

## 1. Introduction

The transition from pregnancy to parenthood is both an exciting and stressful milestone for parents [1]. Postpartum depression (PPD), a depressive disorder occurring within the first year after childbirth, affects approximately 15% of women worldwide [2]. More broadly, anxiety and depressive symptoms either as full syndromal disorders or, more commonly, at a subsyndromal level are among the most prevalent perinatal morbidities, with up to 20% of women experiencing clinically relevant symptoms during pregnancy or in the postpartum period [3,4]. Despite their widespread burden, perinatal mental health disorders remain underrecognized and undertreated, with only 30% of affected women identified in clinical settings and fewer than 7% receiving adequate care [5].

The public health implications of these disorders are substantial. Maternal mental health is increasingly recognized as a key determinant of maternal and neonatal morbidity [3,6,7]. In the United States, mental health conditions have become the leading cause of preventable pregnancy-related mortality across the prenatal, intrapartum, and postpartum periods [8].

Maternal wellbeing shapes not only pregnancy outcomes but also the infant’s long-term development. Maternal behaviors, emotional regulation, and stress physiology during pregnancy influence fetal growth and neurodevelopment [9]. Following birth, sensitive caregiving and co-regulation strategies support the infant’s emerging capacities for emotional and behavioral regulation, empathy, and social functioning [10,11]. Understanding the stress levels and coping strategies of women in early motherhood is therefore important from mental health, developmental, and public health perspectives [12,13,14].

Coping refers to the cognitive and behavioral efforts individuals employ to manage, tolerate, or reduce external and internal demands appraised as stressful [15]. According to Lazarus and Folkman’s transactional model, coping strategies can be broadly categorized into problem-focused coping, which targets the stressor itself, and emotion-focused coping, which aims to regulate emotional responses [15]. Carver’s COPE model further differentiates adaptive strategies such as active coping, planning, and positive reframing from maladaptive ones, including denial, substance use, and behavioral disengagement [16].

In the perinatal context, maladaptive coping strategies have been consistently associated with higher levels of depression and anxiety [17,18] whereas adaptive coping strategies mediate the protective effects of social support on maternal mental health [19].

Recent evidence highlights that maladaptive behaviors such as substance use, self-distraction, and self-blame are positively associated with major postpartum depression in prospective studies, and that coping mediates the relationship between personality traits and depressive symptoms [19,20,21].

Religiosity defined as the degree to which individuals adhere to religious values, beliefs, and practices has also been linked to mental health outcomes [22]. Religiosity may enhance social support through faith communities, provide meaning-making frameworks for stressful events, and promote specific coping strategies such as religious coping [23,24]. However, findings regarding the direct association between religiosity and postpartum depression are inconsistent: some studies report protective effects of religious participation [25], while others find no direct relationship, suggesting religiosity may operate indirectly through coping mechanisms [26,27]. These mixed findings highlight the need to examine both religiosity and coping simultaneously.

Despite robust international literature on perinatal mental health, Eastern Europe—particularly Romania remains understudied. Romania is one of Europe’s most religious countries, where approximately 86% identify as Orthodox Christian [28]. The region is characterized by variability in socioeconomic status, cultural norms, healthcare access, and religiosity levels, yet few studies have simultaneously examined psychological functioning, coping styles, religiosity, and perinatal outcomes among Romanian postpartum women. Bihor and Timiș counties, both in western Romania, were selected for comparison due to their distinct sociocultural profiles. Bihor, bordering Hungary, has a notable Hungarian minority and greater religious diversity, whereas Timiș, home to Romania’s third-largest city (Timișoara), is more urbanized and ethnically homogeneous [29]. These differences may influence coping preferences and psychological adaptation, yet no study has compared perinatal mental health between these regions.

The present study examined coping strategies, mental health symptoms, and religiosity in postpartum women from Bihor and Timiș counties in western Romania. We had three specific objectives: (1) to describe the sociodemographic, obstetric, and psychological characteristics of postpartum women and compare regional patterns; (2) to test associations between coping strategies and depressive/anxiety symptoms; and (3) to investigate whether religious commitment influences coping preferences and moderates coping-symptom relationships. This integrated approach advances understanding of perinatal mental health in Romania and identifies culturally appropriate targets for clinical screening and intervention.

## 2. Materials and Methods

### 2.1. Study Design and Setting

This cross-sectional observational study was conducted in two public maternity hospitals located in the counties of Bihor and Timiș, Romania: Municipal Clinical Emergency Hospital Timișoara and Municipal Clinical Hospital “Dr. Gavril Curteanu” Oradea. Institutional approval was obtained from the Municipal Clinical Emergency Hospital Timișoara authorizing the consultation and use of obstetric and neonatal medical records within Obstetrics-Gynecology Clinic IV. Women were recruited during the early postpartum period, after delivery, while still admitted to the participating hospitals. All data were collected using a structured questionnaire, along with the extraction of obstetric and neonatal information from medical records. Data collection took place between 2024 and 2025 across the two healthcare institutions situated in the western region of Romania.

### 2.2. Participants and Recruitment

Eligible participants were postpartum women who had given birth in one of the two participating hospitals and were able to understand and complete the study questionnaires in Romanian. Women with stillbirth, severe obstetric complications that precluded participation, or documented severe cognitive impairment were not approached. Consecutive eligible women were invited to participate during their inpatient postpartum admission. After providing written informed consent, participants completed a set of self-administered questionnaires, which required approximately 40 min to complete and assessed sociodemographic characteristics, coping strategies, depressive and anxiety symptoms, and religiosity. A total of 201 women were included in the final analytic sample, with 100 recruited from Bihor County and 101 from Timiș County. A total of 201 women were included in the final analytic sample, with 100 from Bihor and 101 from Timiș.

### 2.3. Sociodemographic, Obstetric and Neonatal Data

Sociodemographic information was collected by self-report and included age, living environment (rural/urban), ethnicity, household composition, educational attainment, employment and job category, marital status and perceived family income level. Housing characteristics (satisfaction with housing, number of rooms and number of people in the dwelling) were also recorded.

Obstetric and pregnancy-related variables were abstracted from medical records and supplemented, where necessary, by maternal report. These included anthropometric measures (height, weight at pregnancy onset or delivery, gestational weight gain, body mass index and BMI category), gestational age at first admission, smoking and alcohol use during pregnancy, impaired sleep quality, nausea/vomiting, infections, anemia, miscarriage or bleeding, placental abruption, preterm contractions, pregnancy-induced hypertension, twin pregnancy and surgical interventions during pregnancy. Use of antenatal care and investigations was assessed through the number of antenatal visits and the performance of TORCH testing, first-trimester screening, fetal morphology ultrasound, cervical cultures in the third trimester and Pap smears during pregnancy.

Delivery characteristics included mode of delivery (vaginal vs. cesarean section), medically induced labor, term vs. preterm birth, twin delivery and the presence of recorded delivery complications. Neonatal outcomes comprised gestational age at birth, birth weight and length, Apgar scores at 1 and 5 min and newborn length of stay in intensive care, where applicable. For all variables, the number of missing observations was recorded and reported in the tables; analyses were performed using available cases for each variable.

### 2.4. Psychological Measures

#### Coping Strategies

Coping strategies were assessed using the COPE Inventory, originally developed by Carver, Scheier, and Weintraub [16]. The COPE is a 60-item self-report instrument comprising 15 subscales that assess distinct coping strategies, each rated on a Likert-type scale and summarized as mean scores. The subscales include positive reinterpretation and growth, mental disengagement, expression of emotions, use of instrumental support, active coping, denial, religious coping, humor, behavioral disengagement, restraint coping (abstinence), use of emotional support, substance use, acceptance, suppression of competing activities, and planning. Higher scores indicate greater use of the respective coping strategies [16]. The Romanian translated version of the COPE Inventory, which has previously been used and evaluated in Romanian samples, was administered to participants in the present study [30].

In Romania, the instrument was translated and validated by Crașovan and Sava (2013), who identified a robust four-factor higher-order structure through confirmatory factor analysis (CFA), providing a more stable basis for analysis than individual subscales, which may exhibit lower internal consistency [30]. These higher-order dimensions correspond to problem-focused coping, emotion-focused coping, social support coping, and avoidant coping. In the Romanian validation study, internal consistency for these factors ranged from α = 0.72 to α = 0.84 [30].

In the present sample (N = 201), internal consistency of the COPE higher-order composite scores was acceptable to good, with Cronbach’s alpha values of α = 0.83 (95% CI: 0.80–0.87) for problem-focused coping, α = 0.77 (95% CI: 0.72–0.82) for emotion-focused coping, α = 0.85 (95% CI: 0.82–0.88) for social support coping, and α = 0.79 (95% CI: 0.74–0.83) for avoidant coping.

### 2.5. Depressive Symptoms

Postpartum depressive symptoms were measured with the Edinburgh Postnatal Depression Scale (EPDS), a 10-item self-report instrument assessing symptoms over the previous 7 days. Each item is rated 0–3 (total score range: 0–30), with higher scores indicating greater symptom severity. The Romanian version (EPDS-R) has demonstrated excellent internal consistency in validation studies (α = 0.89) [31]. EPDS scores were analyzed both continuously and categorically using two established cutoffs: EPDS ≥ 10 (possible depression; sensitivity = 0.85, specificity = 0.84) and EPDS ≥ 13 (probable major depression; sensitivity = 0.66, specificity = 0.95) [32]. Internal consistency in the current sample was good (α = 0.83; 95% CI: 0.79–0.86).

Depressive symptoms were also assessed using the Patient Health Questionnaire-9 (PHQ-9), which evaluates nine DSM-based depressive symptoms on a 0–3 scale, for a total score range of 0–27. PHQ-9 scores were analyzed as a continuous variable and were also categorized into minimal, mild, moderate, moderately severe and severe depression, according to established thresholds [33]. The PHQ-9 was originally developed by Robert L. Spitzer et al. [34] during a grant funded by Pfizer Inc. and is freely available for use, reproduction, translation, and distribution without permission. The Romanian version, translated by the instrument’s developers, was obtained from https://www.phqscreeners.com (accessed on 20 January 2026). and is considered the official translated version [34]. Internal consistency for the PHQ-9 was acceptable (Cronbach’s α = 0.82; 95% CI: 0.78–0.86), consistent with previous findings (α = 0.839) [35].

### 2.6. Anxiety Symptoms

Anxiety symptoms were measured with the Generalized Anxiety Disorder 7-item scale (GAD-7). Each item is rated 0–3, yielding total scores from 0 to 21. GAD-7 scores were analyzed as a continuous variable and categorized into minimal, mild, moderate and severe anxiety using standard cut-offs [36]. The GAD-7 was originally developed by Spitzer et al. [36] and is freely available without permission requirements. The Romanian version, translated by MAPI Research Institute, was obtained from https://www.phqscreeners.com (accessed on 15 January 2026). In the present study, the GAD-7 demonstrated excellent internal consistency (Cronbach’s α = 0.91; 95% CI: 0.89–0.93), consistent with previous validation studies (α = 0.92) [36,37].

### 2.7. Religiosity

Religiosity was assessed using the Religious Commitment Inventory-10 (RCI-10; Worthington et al., 2003) [38], a 10-item self-report instrument measuring the degree to which individuals adhere to their religious values, beliefs, and practices in daily life. The scale comprises six intrapersonal religious commitment items and four interpersonal commitment items. Items are rated on a 5-point Likert scale ranging from 1 (“not at all true of me”) to 5 (“totally true of me”) and summed to produce a total score (range: 10–50), with higher scores indicating greater religious commitment.

For the present study, the RCI-10 items were translated into Romanian. While a formal validation study was not conducted prior to data collection, the Romanian version demonstrated excellent internal consistency (α = 0.94; 95% CI: 0.92–0.95), exceeding reliability estimates from the original validation study (α = 0.92–0.98; Worthington et al., 2003) [38] and comparable to other international adaptations including Turkish (α = 0.879; Benk and Budak (2020) [39], Greek (α = 0.869; Satsios, 2016) [40], Farsi (α = 0.85; Hafizi et al., 2016) [41], and Polish (α = 0.82–0.95; Polak & Grabowski, 2017) [42] versions. For descriptive analyses, RCI-10 scores were treated as a continuous variable and categorized into four levels of religiosity (low, low-moderate, moderate, and high) using pre-specified cut-offs.

#### Data Management and Handling of Missing Data

All data were entered into an electronic database and checked for completeness and plausibility. For most variables, analyses were based on complete-case data, with the number of missing values indicated in the tables.

All data were entered into an electronic database and checked for completeness and plausibility. Missing data were minimal across most variables. For most variables, analyses were based on complete-case data, with the number of missing values indicated in the tables. Patterns of missingness were examined descriptively; no systematic patterns were identified based on county, age, education, or obstetric characteristics. Given the low proportion of missing data and absence of systematic patterns, complete-case analysis was deemed appropriate.

### 2.8. Statistical Analysis

Descriptive statistics were used to summarize sociodemographic, obstetric, neonatal, psychological and religiosity variables in the total sample and separately by county (Bihor vs. Timiș). Continuous variables were summarized according to their distributional properties: normally distributed variables (Shapiro–Wilk test *p* > 0.05) were reported as means ± standard deviations (SD), while non-normally distributed variables were reported as medians with interquartile ranges (IQR; 25th–75th percentiles). Categorical variables were reported as counts and proportions *n* (%). Normality assumptions were assessed using the Shapiro–Wilk test prior to selecting appropriate descriptive statistics. Between-county comparisons for continuous variables were performed using independent-samples *t*-tests when distributional assumptions were met; for categorical variables, χ^2^ tests or Fisher’s exact tests were used as appropriate. Two-sided *p*-values < 0.05 were considered statistically significant.

Psychological and religiosity scores (COPE subscales and composites, EPDS, PHQ-9, GAD-7 and RCI-10) were compared between Bihor and Timiș using the same approach. In the pooled sample and within each county, associations between coping dimensions, personality traits, religiosity, and psychological outcomes (EPDS, PHQ-9, and GAD-7 total scores) were examined using Pearson correlation coefficients. Correlation analyses were reported with corresponding r values and *p*-values. The achieved sample size of 201 provides 81% power to detect correlations of r ≥ 0.20 and between-group differences of d ≥ 0.40 at α = 0.05. Post hoc verification confirmed all significant findings represent effect sizes exceeding these thresholds. Multiple regression analyses were conducted to examine the unique contributions of coping strategies to postpartum depressive symptoms (EPDS) while controlling for potential confounders. The regression model included coping dimensions (problem-focused, emotion-focused, social support, and avoidant coping), religiosity (RCI-10), recruitment center (county), and maternal age as predictors. Continuous predictors were standardized (M = 0, SD = 1) to facilitate interpretation and comparison of effect sizes. Model fit was assessed using R^2^ and adjusted R^2^. Regression assumptions (linearity, independence, homoscedasticity, normality of residuals) were verified through diagnostic plots and statistical tests.

To address potential Type I error inflation due to multiple comparisons, we applied two correction methods: (1) Bonferroni correction for family-wise error rate control, and (2) False Discovery Rate (FDR) correction using the Benjamini-Hochberg procedure (Benjamini & Hochberg, 1995) [43]. The Bonferroni-corrected significance threshold was α = 0.05/number of tests within each family of analyses. FDR correction was applied separately to correlation analyses and between-group comparisons. All hypothesis tests were two-tailed with α = 0.05 before correction. Statistical significance was determined based on FDR-corrected *p*-values, with Bonferroni-corrected results reported for comparison as the most conservative approach.

All statistical analyses were performed using RStudio version 4.3.3 (Posit Software, PBC, Boston, MA, USA), employing complete-case data for each variable.

### 2.9. Ethical Considerations

The study protocol was reviewed and approved by the institutional ethics committees of the participating hospitals. All participants received written and verbal information about the study and provided written informed consent prior to enrolment. Participation was voluntary, and data were anonymized prior to analysis to protect participant confidentiality. The Ethics Committee of the Municipal Clinical Emergency Hospital Timișoara, based on the institutional approval issued under Nr. E-5652/14 October 2024, authorizing the consultation and use of obstetric and neonatal medical records within the Obstetrics-Gynecology Clinic IV. The Ethics Committee of the Clinical County Emergency Hospital Bihor, which granted a favorable consultative opinion under Nr. 14136/8 May 2025, permitting access to the hospital database for the doctoral research project.

## 3. Results

Baseline characteristics were largely comparable between counties (Table 1). Mean age at delivery was 29.7 years (SD 5.8), with no significant difference between Bihor (30.1 years) and Timiș (29.4 years; *p* = 0.389). Similarly, living environment, ethnicity, housing satisfaction, household size, marital status, employment categories, and perceived income levels showed no significant regional differences (all *p* > 0.05). Two sociodemographic characteristics differed significantly. Household composition varied by region (*p* = 0.018), with Bihor women more frequently living with husband and children only (50.0% vs. 33.3%), while Timiș women more often lived in multigenerational households (31.3% vs. 16.0%). Education level also differed (*p* = 0.013), with higher education more prevalent in Bihor (62.0% vs. 52.5%) and primary education more common in Timiș (7.9% vs. 1.0%).

Maternal anthropometric characteristics were similar between counties (Table 2). BMI at pregnancy onset (mean 23.6 kg/m^2^, SD 4.3) and BMI at delivery (mean 28.8 kg/m^2^, SD 4.3) showed no significant regional differences. However, gestational weight gain was higher in Bihor (17.1 kg, SD 5.6) compared with Timiș (15.4 kg, SD 5.4; *p* = 0.049). Neonatal outcomes were largely similar between counties. Gestational age at birth, birth weight, and birth length showed no significant differences (all *p* > 0.05). However, Apgar scores were modestly higher in Timiș at both 1 min (9.1 vs. 8.7, *p* = 0.026) and 5 min (9.4 vs. 9.0, *p* = 0.002), though all scores remained within normal ranges (Table 2).

Health behaviors and obstetric complications showed no significant regional variation. Smoking during pregnancy affected 14.9% of women, and impaired sleep quality affected approximately 60%, with similar rates across counties (Supplementary Table S1). Common obstetric complications—including nausea/vomiting (46.8%), anemia (37.8%), infections (16.4%), and miscarriage/bleeding (13.0%)—occurred at comparable frequencies in both regions (all *p* > 0.05).

**Figure 1:**
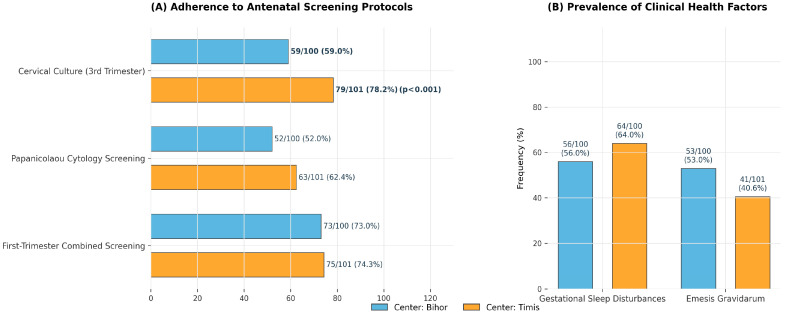
Adherence to Antenatal Screening and Prevalence of Gestational Symptoms in Western Romanian Counties.

Antenatal investigations showed high uptake across regions. TORCH testing was performed in 63.7% of women, first-trimester screening in 73.6%, fetal morphology ultrasound in 85.1%, and Pap smear during pregnancy in 57.2%, with similar frequencies between counties. Women attended a mean of 9.6 antenatal visits in both sites (*p* = 0.955). The only significant difference in antenatal care was the higher frequency of cervical culture performed in the third trimester (Figure 1).

**Figure 2:**
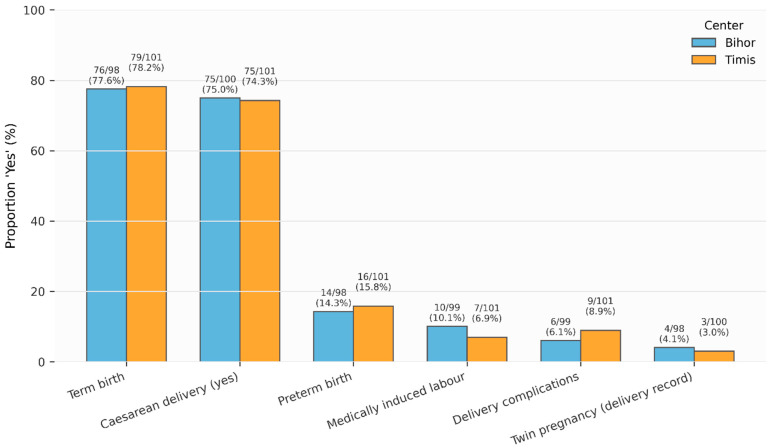
Delivery Mode, Labor Characteristics, and Perinatal Outcomes by County.

Mode of delivery and perinatal outcomes were broadly similar across counties. Term birth occurred in 77.6% of women in Bihor and 78.2% in Timiș. Cesarean delivery was reported in 75.0% of women in Bihor and 74.3% in Timiș. Preterm birth rates were 14.3% in Bihor and 15.8% in Timiș. Medically induced labor occurred in 10.1% and 6.9% of women, respectively. Delivery complications were reported in 6.1% of women in Bihor and 8.9% in Timiș. Twin delivery was uncommon in both regions (4.1% vs. 3.0%). None of these differences reached statistical significance (Figure 2).

**Figure 3:**
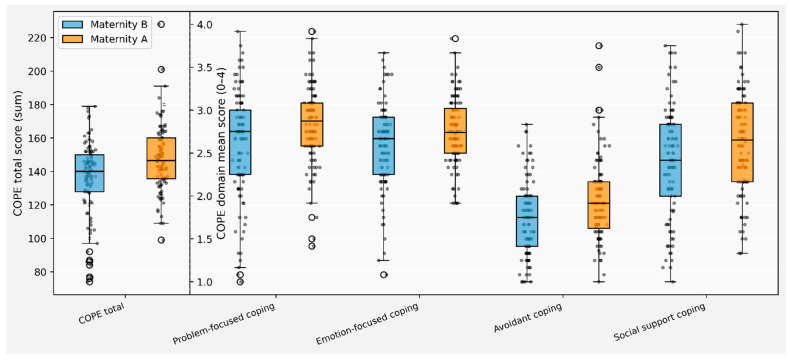
Boxplots of COPE total and domain scores by maternity group. The central line indicates the median; the box represents the interquartile range (IQR; Q1–Q3); whiskers extend to 1.5 × IQR. Points represent individual observations; open circles indicate outliers.

Coping scores were higher in Bihor than in Timiș across all scoring methods. The COPE total score was 141.7 (SD 22.2) overall, with mean values of 147.6 (SD 20.2) in Bihor and 135.8 (SD 22.6) in Timiș (*p* < 0.001), corresponding to COPE mean scores of 2.5 (SD 0.3) and 2.3 (SD 0.4), respectively (*p* < 0.001). Alternative scoring approaches yielded the same pattern of higher coping in Bihor. At the subscale level, significant differences favored Bihor for positive reinterpretation and growth, mental disengagement, instrumental support, active coping, denial, religious coping, planning, problem-focused coping, emotion-focused coping, social support, and avoidant coping (all *p* ≤ 0.039). No significant differences were observed for expression of emotions, humor, behavioral disengagement, abstinence, emotional support, substance use, acceptance, or suppression of competing activities (Supplementary Table S2) (Figure 3). These findings suggest meaningful regional differences in stress management strategies during the perinatal period. The higher overall coping capacity in Bihor, particularly in problem-focused and religious coping domains, may reflect distinct cultural or social support frameworks. The elevated use of active coping, planning, and instrumental support in Bihor indicates a tendency toward solution-oriented approaches to pregnancy-related stressors, which have been associated with better psychological adaptation in perinatal populations. Conversely, the higher avoidant coping scores in Bihor present a more complex picture, as avoidance strategies may serve protective functions in some contexts but can also delay necessary help-seeking behaviors.

EPDS scores were generally low to moderate in the sample, with no statistically significant differences between counties.

The mean EPDS total score was 7.3 (SD 5.1) overall, with 7.5 (SD 4.8) in Bihor and 7.0 (SD 5.5) in Timiș (*p* = 0.535), indicating comparable levels of postpartum depressive symptoms across regions. Using established screening thresholds, 32.8% of women met criteria for possible depression (EPDS ≥ 10), with nearly identical proportions in Bihor (32.0%) and Timiș (33.7%) (*p* = 0.920).

Similarly, 15.4% of the sample scored above the more stringent threshold for probable major depression (EPDS ≥ 13), again with no significant difference between counties (14.0% in Bihor vs. 16.8% in Timiș; *p* = 0.718).

Anxiety symptoms showed a modest difference in mean GAD-7 scores. Mean GAD-7 was 5.1 (SD 4.9) overall, higher in Bihor (5.8 [SD 5.4]) than in Timiș (4.4 [SD 4.2]; *p* = 0.041). However, the distribution of severity levels (minimal, mild, moderate, severe) did not differ significantly between counties (*p* = 0.245).

Religiosity levels were higher in Bihor. The mean RCI-10 total score was 23.1 (SD 10.6) overall, with higher scores in Bihor (25.4 [SD 10.5]) than in Timiș (20.9 [SD 10.2]; *p* = 0.002). The categorization of religiosity also differed (*p* = 0.043): low religiosity was more frequent in Timiș (55.4%), whereas moderate and high religiosity were more prevalent in Bihor (Table 3). The pronounced religiosity differences between counties may have important implications for understanding coping mechanisms and mental health support preferences. Higher religiosity in Bihor aligns with the observed elevation in religious coping strategies, suggesting that faith-based resources may constitute a primary framework for managing perinatal stress in this population. This pattern is theoretically consistent with models proposing that religiosity provides meaning-making structures and social support networks that buffer psychological distress during major life transitions. The relatively lower religiosity in Timiș, despite comparable depression outcomes, suggests that secular coping resources may be equally protective when accessible.

EPDS scores demonstrated strong positive correlations with both general depression (PHQ-9: r = 0.58, *p* < 0.001) and anxiety symptoms (GAD-7: r = 0.61, *p* < 0.001), with similar magnitudes observed in both Bihor and Timiș counties (Table 4). These large effect sizes indicate robust convergent validity between postpartum-specific and general measures of psychological distress.

Associations with coping strategies were considerably weaker and more selective. EPDS was positively associated with social support coping (r = 0.29, *p* < 0.001) and avoidant coping (r = 0.28, *p* < 0.001), with the avoidant coping association most pronounced in Bihor (r = 0.35, *p* < 0.001) compared to Timiș (r = 0.21, *p* = 0.037). Problem-focused and emotion-focused coping showed no significant associations with EPDS in any analysis (both *p* > 0.67). Overall COPE scores showed a weak positive correlation (r = 0.18, *p* = 0.009), though this was not significant within individual counties.

Religiosity showed weak and non-significant associations with EPDS overall (r = 0.12, *p* = 0.091) and within each county (both *p* > 0.05), suggesting religious commitment does not have a direct bivariate relationship with postpartum depressive symptoms in this sample.

Multiple regression analysis examined unique predictors of EPDS scores while controlling for county and maternal age (Table 5). The overall model was significant (R^2^ = 0.196, F(7.190) = 6.614, *p* < 0.001), explaining 19.6% of variance in depressive symptoms.

Three coping strategies emerged as significant independent predictors. Avoidant coping was the strongest risk factor (β = 2.82, 95% CI [1.16, 4.48], *p* < 0.001), with each unit increase associated with a 2.82-point increase in EPDS. Emotion-focused coping showed an unexpected protective effect (β = −2.97, 95% CI [−4.95, −0.98], *p* = 0.004). Social support coping was positively associated with EPDS scores (β = 2.46, 95% CI [1.09, 3.83], *p* < 0.001), likely reflecting help-seeking behavior in distressed women rather than a causal risk factor.

Maternal age showed a small protective effect (β = −0.12, 95% CI [−0.24, −0.002], *p* = 0.047). Critically, county was not a significant predictor after controlling for coping strategies (β = −0.24, *p* = 0.732), suggesting regional differences in mental health outcomes are mediated by differences in coping patterns rather than geographic location per se. Problem-focused coping and religiosity showed no independent effects (both *p* > 0.50).

## 4. Discussion

This cross-sectional study of 201 postpartum women from two public maternity hospitals in Bihor and Timiș, Romania, characterized sociodemographic, obstetric, neonatal, psychological, and religiosity profiles, with a particular focus on coping styles and their associations with depressive and anxiety symptoms. Sociodemographic, obstetric, and neonatal characteristics were broadly comparable between counties. Women in Bihor reported higher coping and religiosity scores and slightly higher mean anxiety levels, whereas depressive symptom severity and the prevalence of possible and probable depression were similar across regions.

To our knowledge, this study represents the first published data on postpartum depression prevalence from Bihor County, addressing a critical gap in the Romanian literature.

### 4.1. Postpartum Depressive and Anxiety Symptoms

Approximately one third of women (32.8%) screened positive for possible depression (EPDS ≥ 10), while 15.4% screened positive for probable major depression (EPDS ≥ 13). The EPDS ≥ 13 prevalence is broadly comparable to commonly reported international estimates of postpartum depression (approximately 17–20%) [44]. In culturally proximate Central and South-Eastern European samples, postpartum depressive symptom prevalence varies by assessment timing, sampling, and thresholds. In Serbia (*n* = 212), EPDS screening at 8 weeks postpartum identified 11% prevalence (EPDS >12), with low education, low financial satisfaction, high-risk pregnancy, and antenatal depressive symptoms as key risk factors [45]. In Slovenia, 16% of nulliparas screened positive at 6 weeks postpartum (EPDS ≥ 10), and postpartum anxiety, partner-attachment anxiety, and employment-related stress were significant predictors after adjustment for prepartum symptoms [46]. Psychometric work in Serbia also indicates substantial symptom burden in postpartum samples (24.8% EPDS ≥ 13) alongside good EPDS reliability (α = 0.83), supporting scale applicability while illustrating heterogeneity in symptom distributions across settings [47].

Similarly, a large epidemiological survey in Hungary (*n* = 1030; 3–26 weeks postpartum) reported 10.81% prevalence using EPDS ≥ 13, with depression during pregnancy as the strongest predictor [48].

In the global literature, Wang et al. reported a pooled postpartum depression prevalence of 17.22% across 565 studies from 80 countries [49]. Within Romania, our EPDS ≥ 13 prevalence is similar in magnitude to data from the same institution reported during the COVID-19 period (18.8%; EPDS >13) [50], whereas higher prevalence has been reported in southeastern Romania when assessed on postpartum day 2 (26.1%), with lower education associated with increased risk [51]. A prospective study from Erzurum, Turkey a culturally traditional, religiously conservative region in eastern Turkey reported PPD prevalence of 17.7% at one week postpartum and 14% at six weeks postpartum [52].

Across the cohort, postpartum depressive symptoms showed strong convergent validity with both general depression and anxiety measures, as reflected by the associations between EPDS and PHQ-9 (r = 0.58, *p* < 0.001) and between EPDS and GAD-7 (r = 0.61, *p* < 0.001). These correlations closely match previous reports, including Zhong et al., who found a correlation of r = 0.52 between PHQ-9 and EPDS [53], supporting the validity of these scales in postpartum populations.

Prior research has shown that depressive symptoms assessed immediately postpartum correlate with symptoms reported several weeks later [54], indicating relative stability in early postpartum mood patterns. Moreover, concerns about infant health and perceptions of infant vulnerability have been identified as contributors to maternal anxiety and stress [55,56]. These findings align with qualitative reports that postpartum anxiety can arise both from uncertainty surrounding childbirth and from concerns about maternal or infant health complications.

### 4.2. Coping Strategies and Depressive Symptoms

One of the central aims of this study was to examine how coping strategies relate to postpartum depressive symptoms. In our sample, avoidant coping showed a positive association with depressive symptoms in bivariate analyses (r = 0.28 overall; r = 0.35 in Bihor; r = 0.21 in Timiș), with the strongest association observed in Bihor, and it also emerged as the most robust coping-related predictor in multivariable models. Comparable patterns have been reported in other European settings. In a large Spanish cohort assessed at 8 and 32 weeks postpartum, passive coping was consistently associated with higher depressive symptoms, and neuroticism was linked to greater passive coping and lower active coping in structural equation models [57]. Similarly, a Polish study using the Mini-COPE measured during pregnancy found that more severe depressive symptoms at six weeks postpartum were associated with helplessness as a coping strategy, particularly among women with lower perceived acceptance/support [58]. This pattern is consistent with evidence that maladaptive coping strategies including denial, behavioral disengagement, self-distraction, and substance-related coping—are associated with higher depressive symptom burden across perinatal populations and may contribute to postpartum depression risk profiles [19,20,21]. Longitudinal evidence indicates that avoidant coping assessed during pregnancy predicts poorer maternal perceptions of the infant and lower caregiving confidence postpartum, and exacerbates the impact of postpartum depression on the mother–infant relationship [59]. Cross-sectional findings further show that avoidant coping is influenced by obstetric and demographic factors and represents a maladaptive coping pattern requiring intervention [17].

Evidence also suggests that coping mechanisms operate within broader vulnerability frameworks. In a large Italian cohort (*n* = 1664), insecure attachment (anxious and avoidant) was associated with higher antepartum depressive symptoms, partly via maladaptive coping. At one week postpartum, anxious attachment showed an indirect association with depressive symptoms through emotional coping, whereas avoidant attachment was not significantly related to depressive symptoms at that early postpartum time point [60].

By contrast, problem-focused coping was not associated with depressive symptoms in either bivariate analyses or multivariable regression. Emotion-focused coping likewise showed no bivariate association, but demonstrated a protective association after adjustment. This pattern is compatible with intercorrelations among coping domains, where multivariable modeling can reveal suppressed protective effects once maladaptive avoidance and other coping dimensions are accounted for, and aligns with prior work showing that “active” coping patterns do not always demonstrate simple unadjusted correlations with depressive severity [61].

Regarding social support coping, we observed a positive association with depressive symptoms, which persisted in regression analyses. This finding should be interpreted alongside the extensive evidence that low perceived support availability is a robust risk factor for postpartum depression. Dennis and Letourneau reported that women who later developed depressive symptoms had lower global and relationship-specific support as early as one week postpartum, with partner-related support emerging as a key predictor of symptoms at eight weeks [62]. Population-based evidence likewise indicates higher odds of postpartum depression among women with moderate or low perceived social support [63]. The divergence from our findings likely reflects measurement differences: the COPE social support dimension captures support-seeking behaviors rather than satisfaction with, or availability of, support. In cross-sectional data, increased help-seeking may therefore function as a marker of distress and unmet needs, rather than implying that seeking support increases depressive symptoms.

### 4.3. Religiosity and Postpartum Symptoms

The present study assessed both religiosity (religious commitment and belief centrality, measured by the RCI-10) and religious coping (behavioral use of religious practices as a coping strategy, measured by a COPE subscale). These constructs showed different patterns of association with mental health outcomes. Women in Bihor reported significantly higher religiosity (RCI-10: 25.4 vs. 20.9, *p* = 0.002) and used religious coping strategies more frequently (COPE religious coping: 3.2 vs. 2.9, *p* = 0.011) compared to women in Timiș. However, religiosity measured by the RCI-10 showed only weak and non-significant associations with depressive symptoms overall (r = 0.12, *p* = 0.091) and within each county. This pattern suggests that religious commitment and belief centrality alone do not directly predict depressive symptom severity in this sample, although they may shape coping preferences such as greater use of religious coping strategies.

Importantly, evidence indicates that religious coping is not uniformly protective and may function differently depending on stressor load and context. For example, a Spanish longitudinal cohort found that higher religious coping predicted higher postpartum depressive symptoms and amplified the association between postpartum pain and depressive symptoms, suggesting that in some settings religious coping may operate as a marker of distress or a maladaptive coping response rather than a buffer [64]. This aligns with the need to interpret coping-based religious measures as potentially reflecting need, strain, or vulnerability, particularly in cross-sectional data.

Prior prospective and large-scale studies suggest that religiosity/spirituality may be protective against postpartum depressive symptoms, but primarily through specific mechanisms rather than belief alone. In a prospective cohort, Mann et al. (2008) [25] found that participation in organized religious activities during pregnancy was strongly protective against postpartum depression, even after controlling for antenatal depressive symptoms and social support, whereas other religiosity/spirituality measures were not significant. Likewise, Cheadle et al. (2018) [26] demonstrated that the association between religiousness/spirituality and lower postpartum depressive symptoms was fully mediated by psychosocial resources (e.g., mastery, self-esteem, optimism), highlighting indirect rather than direct effects. Evidence from culturally specific contexts further indicates that active religious practices—including prayer, scripture recitation, and ritual engagement—can be associated with reduced perinatal anxiety and depression in some populations (Muslim women) [65], while reviews in Latina populations report mixed, context-dependent patterns.

In contrast, religiosity in the present study showed only weak and non-significant associations with postpartum depressive symptoms, despite marked regional differences. This pattern is consistent with prior evidence indicating that intrinsic religious commitment or belief centrality alone does not reliably predict mental health outcomes. Rather, religious participation, psychosocial resources, and the functional role of religious coping behaviors may represent the more proximal pathways linking religion to perinatal mental health. Supporting this distinction, Lara-Cinisomo et al. (2019) [66] found that practical religiosity was associated with situational coping, whereas intrinsic religiosity was not. Qualitative evidence further underscores the importance of sociocultural meaning and context: in rural Ethiopia, postpartum distress was framed around vulnerability and shaped strongly by gender disadvantage and economic hardship, with “cultural dissonance” proposed as a mechanism linking sociocultural norms to distress experiences [67]. Taken together, these findings suggest that religiosity may shape coping preferences (including greater religious coping) without directly determining symptom severity, and that the direction and function of religious coping likely depend on how religious practice interacts with stress exposure, social resources, and local cultural expectations.

## 5. Practical Implications for Prevention and Intervention

The findings of this study have several important implications for clinical practice and public health policy in Romania. First, given the consistent association between avoidant coping and depressive symptoms, brief assessment of coping strategies could be integrated into existing postpartum screening protocols alongside the EPDS. Women identified as relying heavily on avoidant coping (denial, behavioral disengagement, self-distraction) could be prioritized for closer monitoring or early psychological intervention. Such screening could be implemented efficiently in maternity ward settings during the early postpartum admission period, when women are already undergoing routine medical assessments. Second, preventive interventions targeting coping skills should be considered. Antenatal group psychoeducation programs could teach expectant mothers to recognize and modify maladaptive coping patterns before delivery, when stress levels may be lower and motivation for behavior change higher. These programs could focus on practical problem-solving skills, cognitive reappraisal techniques, and appropriate help-seeking behaviors competencies that may prove protective during the postpartum period. Universal prevention approaches of this kind have demonstrated effectiveness in other perinatal populations and may be particularly valuable in public health settings serving diverse socioeconomic groups. Third, for women who screen positive for both elevated depressive symptoms and avoidant coping, targeted brief psychological interventions should be available. Cognitive-behavioral therapy adapted for the postpartum context and problem-solving therapy focused on concrete stressors (sleep deprivation, infant care challenges, social support deficits) represent evidence-based approaches that could be delivered efficiently by trained mental health professionals or adapted as guided self-help interventions in resource-limited settings. Given Romania’s current mental health workforce constraints, low-intensity interventions such as peer support programs led by trained postpartum women or digital interventions may offer scalable alternatives. Fourth, healthcare provider training is essential. Maternity ward staff, primary care physicians, and pediatricians who have regular contact with postpartum women should be trained to recognize signs of avoidant coping and to provide appropriate psychoeducation or referrals. This capacity-building is particularly important in public maternity hospitals, which serve the majority of Romanian women and represent a critical access point for early intervention. Fifth, the observed regional differences in religiosity suggest that culturally informed approaches are necessary. In highly religious regions such as Bihor, interventions may benefit from incorporating or acknowledging religious/spiritual perspectives alongside evidence-based psychological techniques. Collaboration with religious communities may also facilitate help-seeking, reduce mental health stigma, and provide additional social support networks for women experiencing postpartum distress. However, care should be taken to ensure that religious coping supplements rather than substitutes for effective psychological interventions when clinically indicated. Finally, at a systems level, these findings support the development of stepped-care models for perinatal mental health in Romania. Women could be stratified by risk based on coping patterns and symptom severity, with interventions ranging from watchful waiting and psychoeducation (low risk) to specialist mental health referral (high risk). Clear referral pathways connecting maternity services with community mental health resources would facilitate seamless transitions for women requiring more intensive support. Policy recommendations should emphasize integration of perinatal mental health screening and brief coping-focused interventions into Romania’s public health framework, with attention to training, resource allocation, and quality assurance. Future research should prioritize pilot testing of these intervention approaches in Romanian maternity settings, evaluating optimal timing (antenatal versus postnatal), format (individual, group, digital), and intensity. Longitudinal studies are needed to determine whether modifying coping patterns causally reduces subsequent depressive symptom burden and improves maternal and infant outcomes. Such evidence would strengthen the case for systematic implementation of coping-focused interventions within Romanian perinatal care pathways.

## 6. Limitations

Several limitations should also be acknowledged. First, the cross-sectional design precludes causal inferences. It is not possible to determine whether specific coping styles contribute to the development of depressive symptoms, or whether depressive symptoms influence the use of particular coping strategies. Longitudinal studies following women from pregnancy through the postpartum period would be better suited to address temporal relationships. Second, the study is based on a convenience sample from two public hospitals in western Romania and may not be fully representative of all Romanian postpartum women, particularly those delivering in private facilities or in other regions. Third, psychological constructs were assessed using self-report questionnaires, which are susceptible to recall and social desirability bias. Fourth, although the overall sample size was adequate for correlation analyses, it limited statistical power for more granular subgroup analyses and for exploring potential interactions (for example, between coping and socioeconomic characteristics). Fifth, multiple correlations were examined without formal adjustment for multiplicity, so individual *p*-values should be interpreted with caution and the pattern of associations considered as exploratory. Sixth, the RCI-10 has not been formally validated in Romanian populations. While the excellent internal consistency observed in the present study (α = 0.94; 95% CI: 0.92–0.95), which exceeds the original validation and is comparable to international adaptations, provides preliminary evidence of adequate item functioning, future research should conduct comprehensive validation studies including confirmatory factor analysis, test–retest reliability, and convergent/discriminant validity testing in Romanian samples. Additionally, religiosity was assessed using the RCI-10, which measures religious commitment and belief centrality (intrinsic religiosity) but not religious behaviors such as service attendance, prayer frequency, or community involvement. Prior research indicates that religious behaviors and participation may confer mental health benefits through mechanisms distinct from intrinsic belief. While our study included the COPE religious coping subscale (which captures some behavioral aspects of religious practice use), we did not conduct detailed analyses of the association between specific religious coping behaviors and mental health outcomes. Future research should comprehensively assess both intrinsic religiosity and specific religious behaviors (service attendance, prayer frequency, community involvement) and examine their independent and interactive effects on perinatal mental health outcomes.

Despite these limitations, this study contributes novel data on coping styles, depressive and anxiety symptoms, and religiosity among postpartum women in Romania, a region where perinatal mental health remains relatively under-studied. The findings identify avoidant coping as the strongest modifiable risk factor for postpartum depressive symptoms, demonstrate that emotion-focused coping may be protective when other coping strategies are controlled for, and show that regional differences in mental health outcomes are mediated by coping patterns rather than geographic location. The findings further suggest that religiosity influences coping preferences without directly predicting symptom severity. Future research should build on these results using longitudinal designs to establish temporal precedence, broader and more diverse samples to enhance generalizability, detailed examination of religious coping mechanisms, and intervention studies aimed at enhancing adaptive coping and reducing avoidant strategies within Romanian perinatal care pathways.

## 7. Conclusions

This study provides the first comprehensive characterization of coping strategies and their associations with postpartum depression among women from public maternity hospitals in Bihor and Timiș counties. The findings demonstrate that postpartum depression prevalence in these two western Romanian regions aligns with international estimates, that avoidant coping represents the most robust coping-related risk factor for depressive symptoms, and that regional sociocultural differences in religiosity and coping preferences between the two counties do not necessarily translate to differences in mental health outcomes. These results support the integration of brief coping assessment particularly screening for avoidant strategies into postpartum mental health protocols and the development of evidence-based, culturally sensitive interventions targeting maladaptive coping patterns within Romanian perinatal care pathways. With appropriate investment in screening, training, and service development, these two centers and other maternity services across Romania have the opportunity to substantially reduce the burden of postpartum depression through coping-focused prevention and early intervention approaches.

## Figures and Tables

**Table 1:** Sociodemographic characteristics of women included in the study.

Variable	Level	Missing (*n*)	Overall (*n* = 201)	Bihor (*n* = 100)	Timiș (*n* = 101)	*p*-Value
N			201	100	101	
Age at delivery, mean (SD)		3	29.7 (5.8)	30.1 (6.0)	29.4 (5.6)	0.389
Living environment, *n* (%)	Rural	1	88 (44.0)	37 (37.4)	51 (50.5)	0.084
	Urban		112 (56.0)	62 (62.6)	50 (49.5)	
Ethnicity, *n* (%)	Romanian	0	187 (93.0)	90 (90.0)	97 (96.0)	0.079
	Hungarian		6 (3.0)	6 (6.0)	0 (0.0)	
	Roma		3 (1.5)	1 (1.0)	2 (2.0)	
	Other		5 (2.5)	3 (3.0)	2 (2.0)	
Satisfied with housing, *n* (%)	Yes	1	198 (99.0)	98 (99.0)	100 (99.0)	1.000
	No		2 (1.0)	1 (1.0)	1 (1.0)	
Number of rooms in dwelling, mean (SD)		5	3.5 (1.8)	3.3 (1.3)	3.7 (2.1)	0.105
Number of people in dwelling, mean (SD)		4	3.4 (1.6)	3.2 (1.2)	3.5 (1.8)	0.185
Lives with, *n* (%)	Husband	8	67 (34.7)	32 (34.0)	35 (35.4)	0.018
	Husband and children		80 (41.5)	47 (50.0)	33 (33.3)	
	Husband, children and other people		46 (23.8)	15 (16.0)	31 (31.3)	
Education level, *n* (%)	Higher education	0	115 (57.2)	62 (62.0)	53 (52.5)	0.013
	High school		73 (36.3)	33 (33.0)	40 (39.6)	
	Middle school		4 (2.0)	4 (4.0)	0 (0.0)	
	Primary school		9 (4.5)	1 (1.0)	8 (7.9)	
Stressful job, *n* (%)	Yes	31	101 (59.4)	57 (64.8)	44 (53.7)	0.187
	No		69 (40.6)	31 (35.2)	38 (46.3)	
Marital status, *n* (%)	Married	0	179 (89.1)	91 (91.0)	88 (87.1)	0.179
	Divorced		7 (3.5)	5 (5.0)	2 (2.0)	
	Unmarried		14 (7.0)	4 (4.0)	10 (9.9)	
	Widowed		1 (0.5)	0 (0.0)	1 (1.0)	
Job category, *n* (%)	Administrative/office	0	54 (26.9)	24 (24.0)	30 (29.7)	0.259
	Retail & services		36 (17.9)	18 (18.0)	18 (17.8)	
	Unemployed/homemaker/student		35 (17.4)	12 (12.0)	23 (22.8)	
	Education		19 (9.5)	11 (11.0)	8 (7.9)	
	Healthcare		17 (8.5)	11 (11.0)	6 (5.9)	
	Manual/technical/industrial		16 (8.0)	10 (10.0)	6 (5.9)	
	Other		24 (11.9)	14 (14.0)	10 (9.9)	
Perceived family income level, *n* (%)	Income insufficient to cover expenses	0	18 (9.0)	10 (10.0)	8 (7.9)	0.491
	Income sufficient but should be higher		33 (16.4)	20 (20.0)	13 (12.9)	
	Income sufficient to cover expenses		119 (59.2)	55 (55.0)	64 (63.4)	
	Income more than needed		31 (15.4)	15 (15.0)	16 (15.8)	

**Table 2:** Maternal Anthropometrics and Neonatal Outcomes by County.

Variable	Missing (N)	Bihor (N = 100)	Timiș (N = 101)	Total (N = 201)	*p*-Value
BMI at delivery—categorical	11				0.707
Normal weight	—	19 (21.3%)	20 (19.8%)	39 (20.5%)	
Overweight	—	37 (41.6%)	48 (47.5%)	85 (44.7%)	
Obesity	—	33 (37.1%)	33 (32.7%)	66 (34.7%)	
BMI at delivery—continuous	11				0.572
Mean (SD)	—	29.0 (5.1)	28.6 (4.2)	28.8 (4.3)	
Range	—	20.0–48.3	22.1–37.8	20.0–40.9	
BMI at pregnancy onset—continuous	19				0.236
Mean (SD)	—	24.0 (4.6)	23.2 (4.0)	23.6 (4.3)	
Range	—	13.6–40.9	12.8–37.8	12.8–40.9	
Gestational weight gain (kg)	25				0.049
Mean (SD)	—	17.1 (5.6)	15.4 (5.4)	16.1 (5.6)	
Range	—	6.0–30.0	5.0–30.0	5.0–30.0	
BMI at pregnancy onset—categorical	19				0.187
Underweight	—	8 (9.6%)	7 (7.1%)	15 (8.2%)	
Normal weight	—	42 (50.6%)	65 (65.7%)	107 (58.8%)	
Overweight	—	26 (31.3%)	19 (19.2%)	45 (24.7%)	
Obesity	—	7 (8.4%)	8 (8.1%)	15 (8.2%)	
Gestational age at birth (weeks)	6	37.5 (4.4)	38.0 (3.8)	37.8 (4.1)	0.421
Birth weight (g)	4	3201.8 (602.6)	3193.4 (506.5)	3197.5 (554.0)	0.916
Birth length (cm)	4	51.6 (5.2)	50.9 (2.6)	51.2 (4.1)	0.227
Apgar score at 1 min	9	8.7 (1.1)	9.1 (1.0)	8.9 (1.1)	0.026
Apgar score at 5 min	33	9.0 (0.5)	9.4 (0.8)	9.2 (0.7)	0.002
Neonatal intensive care unit stay (days)	165	8.5 (11.8)	6.2 (4.5)	7.6 (9.6)	0.410

**Table 3:** Psychological and religiosity scores.

Variable	Category	Overall (*n* = 201)	Bihor (*n* = 100)	Timiș (*n* = 101)	*p*-Value
Problem-focused coping		2.7 (0.6)	2.8 (0.5)	2.6 (0.6)	0.003
Emotion-focused coping		2.7 (0.5)	2.7 (0.4)	2.6 (0.5)	0.013
Social support coping		2.5 (0.6)	2.6 (0.6)	2.4 (0.6)	0.017
Avoidant coping		1.8 (0.5)	2.0 (0.5)	1.7 (0.4)	<0.001
Rosenberg Self-Esteem Scale					
Rosenberg total score		16.8 (4.4)	16.6 (4.4)	17.0 (4.5)	0.556
Rosenberg self-esteem level	High (30–40)	2 (1.0)	1 (1.0)	1 (1.0)	0.993
	Medium (20–29)	51 (25.4)	25 (25.0)	26 (25.7)	
	Low (10–19)	148 (73.6)	74 (74.0)	74 (73.3)	
EPDS, PHQ-9 and GAD-7					
EPDS total score		7.3 (5.1)	7.5 (4.8)	7.0 (5.5)	0.535
EPDS risk ≥ 10	No	135 (67.2)	68 (68.0)	67 (66.3)	0.920
	Yes	66 (32.8)	32 (32.0)	34 (33.7)	
EPDS risk ≥ 13	No	170 (84.6)	86 (86.0)	84 (83.2)	0.718
	Yes	31 (15.4)	14 (14.0)	17 (16.8)	
PHQ-9 total score		6.0 (4.7)	6.5 (4.7)	5.4 (4.7)	0.111
PHQ-9 severity level	Minimal	88 (43.8)	37 (37.0)	51 (50.5)	0.346
	Mild	73 (36.3)	39 (39.0)	34 (33.7)	
	Moderate	27 (13.4)	17 (17.0)	10 (9.9)	
	Moderately severe	11 (5.5)	6 (6.0)	5 (5.0)	
	Severe	2 (1.0)	1 (1.0)	1 (1.0)	
GAD-7 total score		5.1 (4.9)	5.8 (5.4)	4.4 (4.2)	0.041
GAD-7 severity level	Minimal	112 (55.7)	51 (51.0)	61 (60.4)	0.245
	Mild	57 (28.4)	28 (28.0)	29 (28.7)	
	Moderate	16 (8.0)	10 (10.0)	6 (5.9)	
	Severe	16 (8.0)	11 (11.0)	5 (5.0)	
RCI-10 Religiosity					
RCI-10 total score		23.1 (10.6)	25.4 (10.5)	20.9 (10.2)	0.002
RCI-10 religiosity level	Low	93 (46.3)	37 (37.0)	56 (55.4)	0.043
	Low–moderate	61 (30.3)	33 (33.0)	28 (27.7)	
	Moderate	30 (14.9)	20 (20.0)	10 (9.9)	
	High	17 (8.5)	10 (10.0)	7 (6.9)	

**Table 4:** Correlations with EPDS Total Score.

Pearson Correlation Coefficients with 95% Confidence Intervals							
Psychological Measure	Overall Sample			Bihor Center		Timiș Center	
	N	Overall r [95% CI]	*p*	Bihor r [95% CI]	*p*	Timiș r [95% CI]	*p*
Depressive symptoms (PHQ-9 total)	201	0.58 [0.48, 0.66]	<0.001	0.51 [0.34, 0.64]	<0.001	0.64 [0.51, 0.74]	<0.001
Anxiety symptoms (GAD-7 total)	201	0.61 [0.52, 0.69]	<0.001	0.57 [0.42, 0.69]	<0.001	0.69 [0.57, 0.78]	<0.001
Problem-focused coping	201	−0.02 [−0.16, 0.12]	0.806	−0.15 [−0.33, 0.05]	0.149	0.05 [−0.15, 0.24]	0.633
Emotion-focused coping	201	−0.03 [−0.17, 0.11]	0.677	−0.03 [−0.23, 0.17]	0.752	−0.04 [−0.24, 0.15]	0.676
Social support coping	201	0.29 [0.16, 0.41]	<0.001	0.25 [0.05, 0.42]	0.014	0.32 [0.13, 0.48]	0.001
Avoidant coping	201	0.28 [0.14, 0.40]	<0.001	0.35 [0.16, 0.51]	<0.001	0.21 [0.01, 0.39]	0.037
Religious commitment (RCI-10 total)	201	0.12 [−0.02, 0.25]	0.091	0.03 [−0.17, 0.22]	0.779	0.19 [−0.01, 0.37]	0.058

**Table 5:** Multiple Linear Regression Model Predicting EPDS Scores (N = 201).

Predictor	B	SE	95% CI	β	t	*p*
(Intercept)	8.27	2.60	[3.14, 13.41]	—	3.178	0.002
Coping Strategies						
Avoidant coping	2.82	0.84	[1.16, 4.48]	0.260	3.347	<0.001
Social support coping	2.46	0.69	[1.09, 3.83]	0.298	3.541	<0.001
Emotion-focused coping	−2.97	1.01	[−4.95, −0.98]	−0.258	−2.945	0.004
Problem-focused coping	−0.46	0.81	[−2.07, 1.15]	−0.049	−0.566	0.572
Other Predictors						
Religiosity (RCI-10)	0.02	0.03	[−0.04, 0.09]	0.046	0.669	0.504
County (Timis vs. Bihor)	−0.24	0.70	[−1.63, 1.15]	−0.024	−0.342	0.732
Maternal age (years)	−0.12	0.06	[−0.24, −0.002]	−0.145	−1.998	0.047

## Data Availability

The data presented in this study are available from the corresponding author upon reasonable request. The data are not publicly available due to privacy and ethical restrictions related to sensitive participant information.

## Supplementary Materials

The following supporting information can be downloaded at: https://www.mdpi.com/article/10.3390/jcm15031029/s1, Table S1. Antenatal Care, Obstetric Complications, and Lifestyle Factors by County. Table S2. COPE Inventory Subscales and Composite Scores by County.
