# Supplementary material for: Coping Styles, Postpartum Depression, and Anxiety in Romanian Women: A Cross-Sectional Study Using the Brief COPE Inventory
Source: J Clin Med. 2026 Jan 27;15(3):1029. doi: 10.3390/jcm15031029 (PMC12898781; doi:10.3390/jcm15031029)
Supplement: Supplementary file 1 [file jcm-15-01029-s001.zip › jcm-4077023-supplementary.pdf]

Table S1. Antenatal Care, Obstetric Complications, and Lifestyle Factors by County.

Variable	Bihor (*n* = 100)	Timiș (*n* = 101)	*p*-Value
**ANTENATAL SCREENING & TESTS**			
Fetal morphology ultrasound	87 (87.0%)	84 (83.2%)	0.435
First-trimester screening (12 weeks)	73 (73.0%)	75 (74.3%)	0.826
**Cervical culture in 3rd trimester**	**59 (59.0%)**	**79 (78.2%)**	**<0.001 ***
TORCH tests	64 (64.6%)	64 (64.0%)	0.923
Pap smear during pregnancy	52 (52.0%)	63 (62.4%)	0.140
**OBSTETRIC COMPLICATIONS**			
Surgical interventions during pregnancy	0 (0.0%)	1 (1.0%)	0.323
Twin pregnancy	1 (1.0%)	3 (3.0%)	0.312
Placental abruption (DPPNI)	3 (3.0%)	5 (5.0%)	0.476
Pregnancy-induced hypertension	8 (8.1%)	8 (8.0%)	0.976
Preterm contractions	12 (12.1%)	12 (11.9%)	0.964
Miscarriage/bleeding	16 (16.2%)	10 (9.9%)	0.195
Infections	18 (18.0%)	15 (14.9%)	0.547
Anemia	35 (35.0%)	41 (40.6%)	0.412
Nausea/vomiting	53 (53.0%)	41 (40.6%)	0.078
**LIFESTYLE FACTORS & SYMPTOMS**			
Smoking during pregnancy	13 (13.0%)	17 (16.8%)	0.445

Variable	Bihor (*n* = 100)	Timiș (*n* = 101)	*p*-Value
Impaired sleep quality	56 (56.0%)	64 (64.0%)	0.249

Table S2. COPE Inventory Subscales and Composite Scores by County.

Variable	Category	Overall (*n* = 201)	Bihor (*n* = 100)	Timiș (*n* = 101)	*p*-Value
**COPE—full and composite scores**					
**COPE total (items)**		141.7 (22.2)	147.6 (20.2)	135.8 (22.6)	<0.001
Positive reinterpretation and growth		3.2 (0.6)	3.2 (0.5)	3.1 (0.6)	0.039
Mental disengagement		2.1 (0.6)	2.3 (0.6)	1.9 (0.6)	<0.001
Expression of emotions		2.2 (0.7)	2.3 (0.7)	2.2 (0.7)	0.110
Instrumental support		2.6 (0.8)	2.8 (0.7)	2.5 (0.8)	0.006
Active coping		2.7 (0.7)	2.9 (0.5)	2.6 (0.7)	<0.001
Denial		1.7 (0.6)	1.9 (0.6)	1.6 (0.6)	0.003
Religious coping		3.1 (0.8)	3.2 (0.7)	2.9 (0.9)	0.011
Humor		2.1 (0.8)	2.1 (0.8)	2.0 (0.8)	0.161
Behavioral disengagement		1.7 (0.6)	1.7 (0.6)	1.6 (0.6)	0.237
Abstinence		2.4 (0.6)	2.5 (0.5)	2.3 (0.6)	0.059
Emotional support		2.7 (0.8)	2.8 (0.8)	2.6 (0.8)	0.112
Substance use		1.1 (0.4)	1.1 (0.4)	1.1 (0.4)	0.459
Acceptance		2.5 (0.7)	2.5 (0.6)	2.4 (0.7)	0.107
Suppression of competing activities		2.5 (0.6)	2.6 (0.6)	2.4 (0.7)	0.081
Planning		2.9 (0.7)	3.1 (0.6)	2.8 (0.7)	0.014
